# Large Variations in Malaria Parasite Carriage by Afebrile School Children Living in Nearby Communities in the Central Region of Ghana

**DOI:** 10.1155/2020/4125109

**Published:** 2020-09-22

**Authors:** Evans K. Obboh, Ruth E. Okonu, Linda E. Amoah

**Affiliations:** ^1^Department of Microbiology and Immunology, School of Medical Sciences, University of Cape Coast, Cape Coast, Ghana; ^2^Immunology Department, Noguchi Memorial Institute for Medical Research, University of Ghana, Accra, Ghana; ^3^West Africa Center for Cell Biology of Infectious Pathogens, University of Ghana, Accra, Ghana

## Abstract

**Background:**

Indicators of successful malaria control interventions include a reduction in the prevalence and densities of malaria parasites contained in both symptomatic and asymptomatic infections as well as a reduction in malaria transmission. Individuals harboring malaria parasites in asymptomatic infections serve as reservoirs for malaria transmission. This study determined the prevalence of asymptomatic malaria parasite carriage in afebrile children attending six different schools in two districts, the Cape Coast Metropolitan Assembly (CCMA) and the Komenda Edina Eguafo Abirem (KEEA) of the Central Region of Ghana.

**Methods:**

This cross sectional study recruited afebrile children aged between 3 and 15 years old from six randomly selected schools in the Central Region of Ghana. Finger-pricked blood was collected and used to prepare thick and thin blood smears as well as spot a strip of filter paper (Whatman #3). Nested PCR was used to identify *Plasmodium falciparum*, *Plasmodium malariae*, *Plasmodium ovale*, and *Plasmodium vivax* in DNA extracted from the filter paper spots. The multiplicity of *P. falciparum* infection was determined using merozoite surface protein 2 genotyping.

**Results:**

Out of the 528 children sampled, PCR identified 27.1% to harbor *Plasmodium* parasites in asymptomatic infections, whilst microscopy identified malaria parasites in 10.6% of the children. The overall PCR estimated prevalence of *P. falciparum* and *P. malariae* was 26.6% and 1.3%, respectively, with no *P. ovale* or *P. vivax* identified by PCR or microscopy. The RDT positivity rate ranged from 55.8% in Simiw to 4.5% in Kuful. Children from the Simiw Basic School accounted for 87.5% of all the asymptomatic infections. The multiplicity of *P. falciparum* infection was predominantly monoclonal and biclonal.

**Conclusions:**

The low prevalence of asymptomatic malaria parasite carriage by the children living in the Cape Coast Metropolis suggests that the malaria control interventions in place in CCMA are highly effective and that additional malaria control interventions are required for the KEEA district to reduce the prevalence of asymptomatic malaria parasite carriers. No molecular evidence of *P. ovale* and *P. vivax* was identified in the afebrile children sampled from the selected schools.

## 1. Background

Asymptomatic *Plasmodium* parasite carriage is a dire setback to malaria control and elimination efforts, particularly as they serve as potential reservoirs for malaria transmission [1]. Microscopy, the gold standard technique employed in most malaria-endemic settings for the diagnosis of malaria [2], has a technical detection limit (LoD), which is defined as the lowest number of parasites/*μ*l of blood at which parasites in a blood smear would be detected [3] of between 5 and 10 parasites/*μ*l of blood [4] but a more practical LoD of between 40 and 100 parasites/*μ*l [5]. The wide range observed in the LoD stems mainly from the level of expertise of the microscopist [4, 5]. Majority of asymptomatic malaria infections however are at submicroscopic densities, failing detection by microscopy, making microscopy an inappropriate tool to use for the identification of malaria parasites contained in asymptomatic infections.

Although *Plasmodium falciparum* (*P. falciparum*) is the predominant *Plasmodium* species in the World Health Organization (WHO) Africa Region, that accounts for more than 90% of the malaria-associated morbidity and mortality in this region [6]. *Plasmodium malariae* and *P*. *ovale* have been identified in some sub-Saharan African countries including Ghana at a very low rate [7–11]. The low prevalence of these non-falciparum parasites could, however, be a result of technical difficulties associated with their detection [4, 12]. *Plasmodium vivax* was previously thought to be restricted to Asia and South America due to the absence of the Duffy antigen, which has been indicated as a requirement for erythrocyte invasion by *P. vivax* merozoites [13] among the African population. Interestingly, there are increasing reports of *P. vivax* in West African countries including Benin, Mali, Senegal, and Nigeria [14–16] that call for an increase in the surveillance of all human malaria parasites in malaria endemic countries including Ghana [17]. The WHO recommended treatment for both *P. malariae* and *P. falciparum* is artemisinin combination therapy (ACT), whilst the recommended treatment of *P. ovale* and *P. vivax* requires the supplementation of the ACT with a hypnozoticidal agent such as primaquine (PQ), for the clearance of dormant hypnozoites (radical cure) [18, 19]. It is therefore very important that all carriers of *P. ovale* and *P. vivax* are accurately identified and provided with a radical cure treatment. This study used a sensitive molecular tool to identify and determine the prevalence of four human *Plasmodium species* circulating in afebrile school children living in the Central Region of Ghana.

## 2. Methods

### 2.1. Study Site

**Figure 1:**
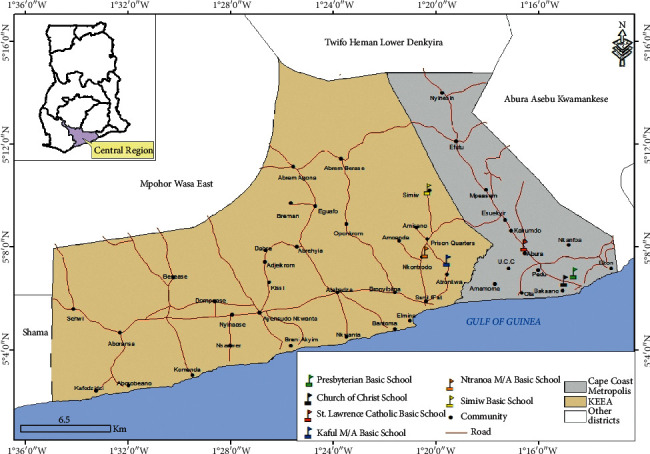
Map of Ghana showing the location of the selected schools within the Central Region created using shapefiles from the Survey Department of the Ghana Statistical Services and ArcMap GIS v10.5 (no administrative permissions were needed to access the shapefiles). Courtesy: Mr. Richard Adade, GIS and Remote Sensing Unit, Department of Fisheries and Aquatic Sciences, Center for Coastal Management, University of Cape Coast, Cape Coast, Ghana.

Six basic schools were selected from two municipalities in the Central Region of Ghana: Presbyterian Basic School (Presby), Church of Christ Basic School (Ewim), and St. Lawrence Catholic Basic School (St Lawrence) from the Cape Coast Municipality (CCMA) and Kuful Basic School (Kuful), Ntranoa Basic School (Ntranoa), and Simiw Basic School (Simiw) from the Komenda Edina Eguafo Abrem District (KEEA) (Figure 1).

The Cape Coast Municipality lies within latitudes 50.07′ to 50.20′ North of the Equator and between longitudes 1°.11′ and 1°.41′ West of the Greenwich Meridian and is bound to the south by the Atlantic Ocean and to the West by the Komenda Edina Eguafo Abrem District.

The Komenda Edina Eguafo Abirem (KEEA) District lies between longitude 1° 20′ West and 1° 40′ West and latitude 5° 05′ North and 15° North.

Vegetation, rainfall, and temperatures in both areas are similar, with the vegetation consisting of mainly secondary forest with thickets and shrubs. The temperature ranges between 24°C and 32°C with relative humidity ranging from 60% to 80%. The peak rainfall season is between May to June and the peak malaria season from June to August [20].

### 2.2. Sample Collection and Processing

The Institutional Review Board of the Noguchi Memorial Institute for Medical Research granted ethical clearance for this study (IRB Approval #: 050/12-13). Permission to conduct this study was also obtained from the KEEA and CCMA Municipal Directors of Education. Parents and guardians, teachers of the school children as well as the school children were engaged during a number of encounters at each school premise where the study's aims and objectives were explained. Willing parents/guardians provided written parental consent for the children prior to being recruited into the study, and children aged 12 years and above were also made to endorse assent forms.

All children aged between 3 and 15 years whose parent/guardian endorsed the parental consent forms were recruited into the cross-sectional study. Two drops of finger-pricked blood (∼100 *µ*l) were collected from 528 afebrile children in October 2017. The blood was used to prepare thick and thin blood smears and dried blood spots (DBS) [21] as well as to load a CareStart^™^ malaria HRP2 rapid diagnostic test kit (RDT) following the manufacturer's instructions. Each DBS was stored individually in a Ziploc bag containing a desiccant.

### 2.3. Microscopy

Thick and thin blood smears were processed for malaria parasite identification and quantification according to the WHO protocol [12, 22] as previously described [11]. Two independent microscopists read the slides, and discordance in calling a positive or negative smear was resolved by a third microscopist.

### 2.4. DNA Extraction

Genomic DNA was extracted from DBS using the Chelex extraction method [23, 24]. Briefly, two 3 mm disks were punched out of each DBS into a 1.5 ml microfuge tube containing 1 ml of 1X phosphate-buffered saline (PBS, pH 7.4) supplemented with 50 *µ*l of a 10% saponin solution. After an overnight incubation, the PBS/saponin solution was decanted and the discs were washed with 1 ml of ice-cold PBS and left at 4°C. After a 30-minute incubation, the tubes were centrifuged at 10,000 ×g for 30 seconds and the supernatant was discarded. A freshly prepared solution of 20% Chelex-100 (60 *µ*l) and 140 *µ*l of distilled water were then added to each tube. The tubes were heated for 10 minutes at 95°C and then centrifuged at 13,000 rpm for 6 minutes. The supernatant (∼120 *µ*l) containing the extracted DNA was then transferred into a new sterile labeled 0.5 ml microfuge tube and either used immediately or stored at −20°C for later use.

### 2.5. *Plasmodium* Species (*P. falciparum*, *P. malariae*, *P. ovale*, and *P. vivax*) Identification

A nested PCR protocol adapted from Snounou et al. [25] and similar to Amoah et al. [11] was used to amplify specific regions of the 18s rRNA gene from the four different parasite species. Briefly, the primary reaction contained 80 nM of the genus-specific primer rPLU5 and rPLU6 (Additional file Table S1) and between 30 and 50 ng (5 *µ*l) of DNA template supplemented with 1X PCR buffer, 167 nM dNTPs, 2.5 mM MgCl_2_, and 1 U of OneTaq DNA polymerase. The secondary reaction used 0.5 *µ*l of the primary reaction product as template and either of the four sets of species-specific primers (Additional file Table S1) 133.3 nM of rFAL1/rFAL2 (*P. falciparum*-specific), 333.33 nM of rMAL1/rMAL2 (*P. malariae*-specific), rOVA1/rOVA2 (*P. ovale*-specific), and rVIV1/rVIV2 (*P. vivax*-specific) in addition to a reaction mixture similar to that described for the primary reaction (a representative image of the *Plasmodium* species PCR products is in Additional file Figure S1). The positive controls used in the *Plasmodium* speciation reaction include gDNA from 3D7 (MRA-102, *P. falciparum control* (205 bp)), gDNA from a *P. ovale* field isolate (787 bp), gDNA from a Pf/Pm mixed infection (field isolate) used as an in-house *P. mal* control (144 bp), and gDNA from *P. vivax* (obtained as part of the 2019 WHO NAAT EQA Scheme (117 bp)).

### 2.6. Determination of Multiplicity of Infection (MOI)

The MOI of *P. falciparum* infection was determined by genotyping the msp2 locus using nested PCR as previously described (21, 27). Briefly, the two allelic families (FC27 and IC3D7) of the msp2 gene were differentiated in the central polymorphic region. Block 3 of the msp2 gene was amplified from about 50 *µ*g of parasite DNA using the M2-OF and M2-OR primer set (Additional file Table S1) in a primary reaction mixture containing 200 nM of dNTP, 2 mM of MgCl_2_, and 0.5 U of OneTaq DNA Polymerase (New England Biolabs MA, USA). The primary PCR product (0.5 *µ*l) was then amplified using two sets of primers, N5 rev and S1w-f (amplifies IC3D7 alleles) and M5 rev and S1w-f (amplifies FC27 alleles) (200 nM of each) in a PCR mixture containing 200 nM of dNTP, 1.8 mM of MgCl_2_, and 0.5 U of OneTaq DNA Polymerase. Positive control samples including gDNA extracted from the K1 (MRA-159G) and 3D7 (MRA-102G) parasite strains and a negative no template control samples were included in all PCR amplifications.

### 2.7. Data Analysis

The prevalence of asymptomatic infections was calculated as the proportion of children in each school who tested positive for malaria parasites (irrespective of the species contained in the infection).

Multiplicity of infection was defined as the number of different *P. falciparum* parasite clones detected in a sample. Such that samples containing a single amplified product after msp2 PCR were considered as clonal, samples containing 2 amplified products were considered biclonal, and those with more than two amplified products were considered as multiclonal.

Statistical analysis was performed using GraphPad Prism version 5 (GraphPad Software, Inc.) and IBM SPSS version 22. The Kruskal–Wallis test followed by Dunn's multiple comparison was used to determine significant differences between the median age of the children, RDT positivity rate, parasite density, and the prevalence of malaria parasites across the different schools. Descriptive statistics including mean and its 95% confidence interval (95% CI), frequency (estimated as a percent), and Pearson chi-square analysis was used to identify significant differences in the distribution of males in the different schools. *P* values of less than 0.05 were considered statistically significant.

## 3. Results

### 3.1. Demographic Characteristics of Study Participants

The study population comprised of 528 children between 3 and 15 years (Table 1) recruited from six schools in the Central Region of Ghana. Significant differences were observed in the distribution of males in the six schools (Pearson's chi-square *P*=18.44, *P*=0.002), with the proportion of males in the study population ranging between 33% (33/100) in children from St. Lawrence and 57.5% (69/120) in children from Simiw. Children from Ewim, Simiw, and Presby Basic schools had similar ages, whilst children from Ntranoa Basic school were significantly older than children from all the other schools (Dunn's multiple comparison test, *P* < 0.001), and children from Kuful Basic school were significantly younger than children from all the other schools (Dunn's multiple comparison test, *P* < 0.05).

### 3.2. Parasite Prevalence

Samples from all the 528 children were tested for the presence of *Plasmodium* infections using microscopy, an HRP2-based malaria RDT and PCR.

#### 3.2.1. Microscopy

Malaria parasites were identified in all but one (Presby Basic School) of the schools, with *P. falciparum* monoinfections accounting for 95.9% of the infections and *P. malariae* accounting for the remaining 4.1% (Table 2). Children from the Simiw Basic School had more than 50% of the microscopic density infections identified in the study. *Plasmodium malariae* was identified in children from only two schools, Simiw and Ewim (Table 2).

#### 3.2.2. Rapid Diagnostic Test (RDT)

The HRP2-based RDT kits provided an estimate of the positivity rates for only *P. falciparum* infections. The overall RDT positivity rate amongst the entire population of school children was 21.2% (112/528). Children from Simiw had the highest RDT positivity rate of 55.8% (67/120), which was significantly higher than the positivity rates detected in children from all the other schools (Dunn's multiple comparison test, *P* < 0.001). Two schools, Presby and Ewim, had RDT positivity rates below 5% and three schools (St Lawrence, Ntranoa, and Kuful) had positivity rates of between 11% and 20%, but the differences between the positivity rates in the five schools were not statistically significant (Dunn's multiple comparison test, *P* > 0.05) (Table 2).

### 3.3. Molecular Detection

#### 3.3.1. *Plasmodium* Species Identification

The overall prevalence of asymptomatic children estimated by PCR was 27.1% (143/528), which included three children harboring *P. malariae* as monoinfections and four children harboring both *P. falciparum* and *P. malariae* as mixed-species infections. *Plasmodium falciparum* was identified in 26.5% (140/528) of the children. No *Plasmodium* parasites were identified in children from the Ewim and St. Lawrence Basic Schools (Table 2). The highest prevalence of children with asymptomatic *P. falciparum* infections was identified in Simiw, where 87.5% (105/120) of the children carried *P. falciparum* parasites (Table 2). *Plasmodium falciparum* parasite carriage in Simiw was significantly higher (Dunn's multiple comparison, *P* < 0.001) than *P. falciparum* carriage identified in children from all the other five Basic Schools. No significant difference was observed in the prevalence of *P. falciparum* parasite carriage in four of the Basic Schools: Presby, St Lawrence, Ewim, and Ntranoa (Dunn's multiple comparison, *P* > 0.05). However, parasite carriage in Kuful was significantly higher than parasite carriage in Presby (Dunn's multiple comparison, *P* < 0.05) but similar to that identified in Ntranoa (Dunn's multiple comparison, *P* > 0.05).

A low prevalence of *P. malariae* was identified (Mann–Whitney test, *P*=0.412) in children from two of the Basic Schools, Ntranoa at 2.9% (2/70) and Kuful at 5.6% (5/90). The five *Plasmodium malariae* infections identified in Kuful were primarily (80%) contained in mixed infections with *P. falciparum*, with only 1/5 (20%) identified as a *P. malariae* monoinfection. In Ntranoa, both (100%) of the *P. malariae* infections were identified as monoinfections. No molecular evidence of *P. vivax* or *P. ovale* was identified in any of the children screened (Table 2).

#### 3.3.2. Comparison of the Three Diagnostic Tools

**Figure 2:**
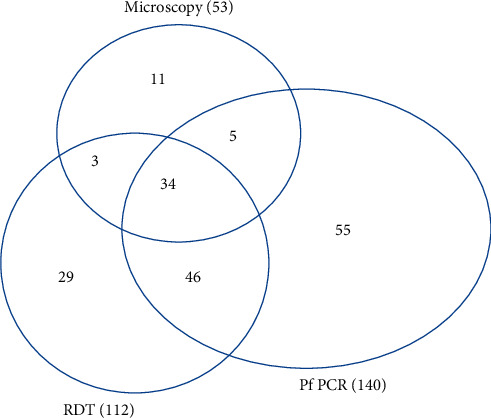
*Plasmodium falciparum* infection detected by multiple tools. A Venn diagram illustrating the results of *P. falciparum* identification by nested PCR and an HRP2-based malaria RDT. The number of positive samples identified by each test is listed in parentheses after the test name.

There were 34 samples that tested positive for *P. falciparum* by all the three tests, microscopy, RDT, and PCR (Figure 2). The RDT analysis identified 46 samples that were positive by PCR, which were missed by microscopy. Microscopy identified 5 samples that were positive by PCR, which were missed by RDT. There were 3 samples that tested positive by both microscopy and RDT but were not detected by PCR.

#### 3.3.3. Sensitivity and Specificity of Diagnostic Tests

**Figure 3:**
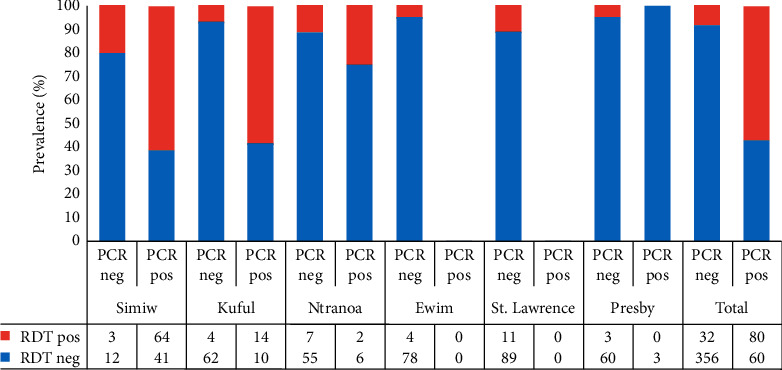
Site-specific sensitivity and specificity of the RDT. A bar graph representing the specificity and sensitivity of the RDT results relative to *P. falciparum* PCR. PCR neg, sample tested negative by PCR; PCR pos, sample tested positive by PCR; RDT neg, sample tested negative by rapid diagnostic test (HRP2); RDT pos, sample tested positive by rapid diagnostic test (HRP2); PCR, polymerase chain reaction. The data in the table represent exact counts.

When RDT results were compared to *P. falciparum*-specific PCR results, a sensitivity of 57.1% (80/140) and a specificity of 91.8% (356/388) were obtained (Figure 3). The highest sensitivity of the RDT kit of 61% (64/105) was obtained in Simiw and the lowest sensitivity was observed in Presby Basic School where none of the PCR-positive samples tested positive by RDT. The RDT results from the Presby and Ewim Basic Schools had specificities of slightly greater than 95%, whilst RDT results from Simiw yielded the lowest specificity of 80% (Figure 3).

**Figure 4:**
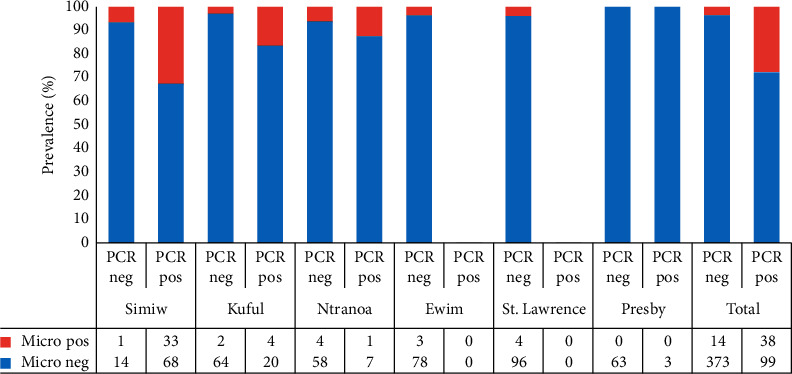
Site-specific sensitivity and specificity of microscopy. A bar graph representing the specificity and sensitivity of the *P. falciparum*-specific microscopy results relative to *P. falciparum*-specific PCR. PCR neg, sample tested negative for *P. falciparum* by PCR; PCR pos, sample tested positive for *P. falciparum* by PCR; micro neg, sample that did not contain *P. falciparum* by microscopy; micro pos, sample that tested positive for *P. falciparum* by microscopy. The data in the table represent exact counts.

The sensitivity of microscopy was relatively low, 27.7% (38/137) overall and ranged from 32.7% in Simiw to 12.5% in Ntranoa (Figure 4). The specificity of microscopy was 96.4% (363/384) overall and ranged from 93.3% in Simiw to 100% in children from the Presby Basic School. There were 14 microscopy-positive samples that tested negative for *Plasmodium* parasites by PCR (Additional file Table S2). These samples were mostly from low-density infections and had a parasite density range of 36 parasites/*µ*l to 160 parasites/*µ*l.

#### 3.3.4. Multiplicity of *P. falciparum* Infection

**Figure 5:**
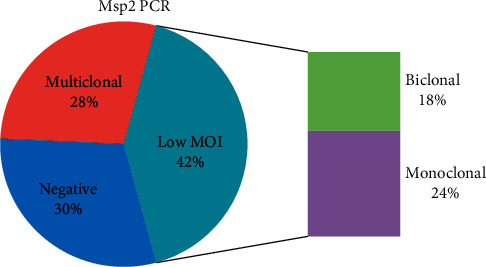
Multiplicity of Infection. A bar of pie chart illustrating the msp2 PCR results. The *P. falciparum*-positive samples were subjected to msp2 PCR to determine the multiplicity of infection (MOI). Samples that did not yield any PCR amplicon were classified as negative. Those that yielded more than two amplicons were classified as multiclonal and those that yielded one (monoclonal) and two (biclonal) classified as containing infections with low MOI. The numbers in the figure are as a percent of the 140 *P. falciparum*-positive samples.

Genotyping of *P. falciparum*-positive samples at the merozoite surface protein 2 (msp2) locus identified 70% (98/140) of the samples as positive. A majority (59.2%, 58/98) of the msp2-positive samples contained infections with low multiplicity of infection (mono and biclonal infections). Monoclonal infections represented 56.9% (33/58) of the low MOI infections. Overall, monoclonal infections represented 33.7% (33/98) of the msp2-positive samples and 23.6% of the *P. falciparum*-positive samples whilst biclonal samples represented 25.5% (25/98) and 17.9% of the msp2- and the *P. falciparum*-positive samples, respectively (Figure 5). All the four *P. falciparum*/*P. malariae* coinfected samples identified by *Plasmodium* species-specific PCR were amongst the msp2-positive samples whilst all the three *P. malariae* monoinfected samples tested negative after msp2 amplification (Figure 5).

## 4. Discussion

Infections containing any of the human malaria parasites including *P. falciparum*, *P. malariae*, *P. ovale*, and *P. vivax* can present as asymptomatic infections [26]. A large proportion of asymptomatic malaria infections are at submicroscopic densities and not detected by microscopy [11]. More importantly under certain conditions, asymptomatic infections can progress to acute disease [27]. *Plasmodium falciparum* is known to cause the most lethal form of malaria; however, both *P. ovale* and *P. malariae* are capable of causing fatal disease, with reports suggesting an association of *P. malariae* with hematological morbidity [28] and *P. ovale* with acute respiratory distress syndrome [29]. It is therefore necessary that both symptomatic and asymptomatic malaria infections are actively detected [30] and effective treatment regimens administered to clear all the infecting malaria parasites [31]. This study used PCR, RDT, and microscopy to determine the presence and prevalence of malaria parasites carried as asymptomatic infections in children living in two districts of the Central Region of Ghana.

Children from the KEEA district generally had higher parasite prevalence than children from the CCMA, with children from the Simiw Basic School accounting for almost 90% of the total *Plasmodium* infections identified in this study. A possible reason for the lower parasite prevalence in the CCMA compared with the KEEA district is that the CCMA is the district capital of the Central Region and is more urbanized than the KEEA, which comprises of more semirural settlements [32]. The results from this study support the observation that malaria parasite prevalence in rural settings is usually much higher than in semirural settings [33]. The very high prevalence of malaria parasites in Simiw may be as a result of the large number of swamps [34] as well as houses without net screens covering the doors and windows [35], which are important malaria vector control tools [36, 37]. Entomological inoculation rates for the various communities could help in explaining the differences in parasite prevalence rates identified in the different schools; however, such data are not available. These results are contrary to a report from the Eastern Region of Ghana where parasite prevalence in seven closely linked communities was similar [11], but support a recent report from Laos, SE Asia, where high heterogeneity in the distribution of malaria parasites in closely linked communities was identified [38].

The presence of asymptomatic infections containing *P. malariae* supports findings from a number of studies from Ghana that have reported the presence of *P. malariae* in some infections [25, 39]. No *P. vivax* or *P. ovale* was identified by PCR and microscopy. The absence of *P. vivax* is not surprising as until recently, no *P. vivax* infections were identified in sub-Saharan Africa due to the absence of the Duffy antigen on erythrocytes in this population [17] and there have been no reports to date of *P. vivax* in Ghana.

The presence of asymptomatic infections containing *P. malariae* was not surprising as a number of studies from Ghana have reported an increase in the prevalence of *P. malariae* infections [7, 11, 40]. The absence of *P. vivax* in this study population is not surprising as there have been no reports of the presence of *P. vivax* in Ghana to date. However, *P. vivax* has recently been identified in a number of sub-Saharan African countries including Mali, Senegal, Nigeria, and Cameroon [14, 15, 41, 42] despite the absence of the Duffy antigen, which is required for *P. vivax* invasion into erythrocytes. This finding in neighboring countries calls for enhanced surveillance of *P. vivax* in Ghana.

Although the HRP2-based malaria RDT kits are the most sensitive and widely used RDT for malaria diagnosis, they are noted to have low specificity due to the persistence of the HRP2 antigen in the blood. The HRP2 antigen has been found in samples several days to weeks after the treatment of malaria and clearance of the parasites [43, 44]. Any child with a false-positive RDT result most likely had recently been successfully treated for malaria but had some HRP2 antigen persisting in the blood. This assumption is clearly supported in Simiw where parasite prevalence was the highest. The high prevalence of malaria parasites in high malaria trnamission settings suggests the children are often exposed to malaria parasites and subsequently have high concentrations of the HRP2 antigen persisting in circulation. The high HRP2 antigen concentrations in people living in high transmision settings can result in high false-positive RDT results and thus low RDT specificity. Similarly, sites with low parasite prevalence had very high RDT specificities, most likely due to infrequent encounters with the malaria parasite and thus low concentrations of the HRP2 antigen. The presence of samples that tested positive for *P. falciparum* by PCR but tested negative by RDT could be a result of low parasite densities, which are common during the off-peak malaria season. Sample collection for this study was conducted during the off-peak malaria season where parasite prevalence and densities are expected to be low, especially in the Central Region of Ghana [45] and from asymptomatic children who are known to carry submicroscopic densities of parasites [46]. The accuracy and sensitivity of malaria RDT kits are mostly high at high parasite densities, with a threshold set at 200 parasites per microliter [47]. The presence of parasites with deletions in the HRP2 gene can also result in false-negative RDT results [48]; however, this was not explored in this study.

Microscopic evaluation of thick blood smears for microscopy is subjective and accuracy reduces as the parasite density of the sample reduces [49] and also when non-*falciparum* species are encountered [50, 51], especially as these non-*falciparum* species are usually encountered at a low density and or in a mixture with *P. falciparum* [52, 53]. A few *P. falciparum* infections were misclassified as *P. malariae*, likely due to inadequate smear preparation or the presence of some unanticipated mature forms of *P. falciparum* in the smear, which prevented the microscopists from efficiently identifying the correct *Plasmodium* species. This is a major concern for malaria microscopy that has been previously reported in countries including Ghana [11, 50] and suggests that malaria microscopists together with the technicians who prepare the thick and thin films require frequent refresher training sessions to ensure high levels of accuracy in the reporting of malaria microscopy data. The low sensitivity of microscopy identified in this study supports numerous earlier reports and is likely due to the low-density parasites normally associated with asymptomatic infections [46]. Although PCR is a highly sensitive tool that can detect *Plasmodium* parasites at very low densities [25], it has been found under instances of low parasite density to occasionally have reduced accuracy [54].

The reduced amplification efficacy obtained after performing the msp2 genotyping reactions relative to the *Plasmodium falciparum* 18S rRNA gene amplification in this study could be due to the low parasite densities contained in the samples. Secondly, the msp2 locus is a single copy locus relative to the 18s rRNA gene which is a multicopy locus [21], which would require higher concentrations of template for amplification. An earlier study conducted amongst uncomplicated malaria patients from the Central Region of Ghana identified the amplification efficiency of msp2 genotyping to be higher than that reported in this study [55]. The discordance in the reports could be due to symptomatic malaria patients most likely harboring higher parasite densities than afebrile children.

Ghana is still in the WHO malaria control phase; as such, most malaria control interventions target the reduction of clinical malaria, with very little attention paid to asymptomatic manifestations of the disease [56]. Asymptomatic malaria parasite carriage can maintain malaria transmission in a community [57] and has the potential to impair brain development in children as well as increase the vulnerability of the host to other diseases [58]. Policymakers in malaria-endemic countries such as Ghana should consider asymptomatic parasite carriage as a major obstacle to the process of achieving malaria elimination. Community-level awareness of asymptomatic parasite carriage and its negative implications on malaria control as well as the requirement to completely clear all parasites infecting symptomatic and asymptomatic malaria patients should also be advocated for [31].

## 5. Limitations

This study focused only on children, who are the most likely to be affected by malaria episodes due to them having a less matured immune system [59] as well as playing for long hours outdoors amongst others [60].

## 6. Conclusions

The low prevalence of asymptomatic malaria parasite carriage in the Cape Coast Metropolis suggests that malaria control interventions in place in CCMA are highly effective. Similarly, additional malaria control interventions are required for the KEEA district to reduce the prevalence of asymptomatic malaria parasite carriers.

## Figures and Tables

**Table 1:** Demographic characteristics of enrolled children.

	Presby (66)	St. Lawrence (100)	Ewim (82)	Ntranoa (70)	Kuful (90)	Simiw (120)	Total (528)
Sex (M/F)	31/35	33/67	43/39	26/44	35/55	69/51	237/291
Age (years)							
Mean (95% CI)	9.33 (8.9–9.8)	11.17 (10.8–11.5)	8.28 (7.6–8.9)	12.8 (12.5–13.1)	7.07 (6.7–7.5)	8.27 (7.8–8.8)	9.35 (9.1–9.6)
Min-max	4–13	7–15	3–13	9–14	5–12	4–13	3–15

M, male; F, female; N, total number of positive samples; RDT, PfHRP2-based malaria rapid diagnostic test kit; 95%CI, 95% confidence interval; Min, minimum value; Max, maximum value. The schools are listed with the total number of children in parentheses.

**Table 2:** Parasite prevalence.

	Presby (66)	St. Lawrence (100)	Ewim (82)	Ntranoa (70)	Kuful (90)	Simiw (120)	Total (528)
Microscopy							
Pf	0	4	3	5	6	35	53
Pm			1			2	3
RDT	3	11	4	9	18	67	112
PCR							
Pf	3	0	0	8	24	105	140
Pm	0	0	0	2	5	0	7

RDT, HRP2-based rapid diagnostic test kit; Pf, *P. falciparum* detected by PCR; Pm, *P. malariae* detected by PCR. The numbers in the table represent the number of samples that tested positive for each test.

## Data Availability

The datasets used and/or analysed during the current study are available in the document and its supporting files.

## Abbreviations

*P. falciparum*: *Plasmodium falciparum*
*P. malariae*: *Plasmodium malariae*
*P. ovale*: *Plasmodium ovale*
*P. vivax*: *Plasmodium vivax*
HRP2: Histidine-rich protein 2
RDT: Rapid diagnostic test
PCR: Polymerase chain reaction.

## Supplementary Materials

Supplementary Materials Additional file Table S1: primer details. Information on all the primers used in the study. Additional file Figure S1: representative images of *P. falciparum*, *P. malariae*, *P. ovale*, and *P. vivax* PCR products. Additional file Table S2: details of the false-positive samples.
