# Supplementary material for: Large Variations in Malaria Parasite Carriage by Afebrile School Children Living in Nearby Communities in the Central Region of Ghana
Source: J Trop Med. 2020 Sep 22;2020:4125109. doi: 10.1155/2020/4125109 (PMC7528039; doi:10.1155/2020/4125109)
Supplement: Supplementary Materials — Additional file Table S1: primer details. Information on all the primers used in the study. Additional file Figure S1: representative images of P. falciparum, P. malariae, P. ovale, and P. vivax PCR products. Additional file Table S2: details of the false-positive samples. [file 4125109.f1.zip › 4125109.f1/22.8.2020 Sch Screen Tab S2.docx]

Additional file Table S2. Details of samples false positive microscopy samples

| ID | AGE | SEX | RDT | PD |
| --- | --- | --- | --- | --- |
| E 019 | 8 | M | N | 160 |
| E 065 | 10 | F | N | 80 |
| E 072 | 10 | F | P | 80 |
| K 018 | 5 | F | N | 160 |
| K 072 | 7 | F | N | 36 |
| N 015 | 13 | F | N | 48 |
| N 021 | 14 | M | P | 48 |
| N 044 | 13 | M | P | 40 |
| N 052 | 14 | F | N | 80 |
| S 038 | 6 | F | N | 132 |
| SL 043 | 12 | F | N | 80 |
| SL 063 | 9 | F | N | 40 |
| SL 073 | 11 | F | N | 36 |
| SL 077 | 11 | M | N | 80 |

14 samples were identified as containing *P. falciparum* by microscopy but tested negative by *P. falciparum* specific PCR. E, Ewim; K, Kuful; N, Ntranoa; S, Simiw; SL, St. Lawrence; M, male; F, female; PD, parasite density measured as parasite per microliter
