# Supplementary material for: Large Variations in Malaria Parasite Carriage by Afebrile School Children Living in Nearby Communities in the Central Region of Ghana
Source: J Trop Med. 2020 Sep 22;2020:4125109. doi: 10.1155/2020/4125109 (PMC7528039; doi:10.1155/2020/4125109)
Supplement: Supplementary Materials — Additional file Table S1: primer details. Information on all the primers used in the study. Additional file Figure S1: representative images of P. falciparum, P. malariae, P. ovale, and P. vivax PCR products. Additional file Table S2: details of the false-positive samples. [file 4125109.f1.zip › 4125109.f1/22.8.2020 Sch Screening Tab S1.docx]

Additional file Table S1. Primer details. Information on all the primers used in the study

| Species | Primer | Primer Sequence (5’-3’) |
| --- | --- | --- |
| ***Plasmodium spp*** | rPLU6 | TTA AAA TTG TTG CAG TTA AAA CG |
|  | rPLU5 | CCT GTT GTT GCC TTA AAC TTC |
| *P. falciparum*  *(205 bp)* | rFAl1 | TTA AAC TGG TTT GGG AAA ACC AAA TAT ATT |
|  | rFAL2 | ACA CAA TGA ACT CAA TCA TGA CTA CCC GTC |
| *P. malariae*  *(145 bp)* | rMAL1 | ATA ACA TAG TTG TAC GTT AAG AAT AAC CGC |
|  | rMAL2 | AAA ATT CCC ATG CAT AAA AAA TTA TAC AAA |
| *P. ovale*  *(800 bp)* | rOva1 | ATC TCT TTT GCT ATT TTT TAG TAT TGG AGA |
|  | rOva2 | GGA AAA GGA CAC ATT AAT TGT ATC CTA GTG |
| *P. vivax*  *(120)* | rViv1 | CGC TTC TAG CTT AAT CCA CAT AAC TGA TAC |
|  | rVIV2 | ACT TCC AAG CCG AAG CAA AGA AAG TCC TTA |
| **msp2** |  |  |
| Primary | M2- OF: | ATGAAGGTAATTAAAACATTGTCTATTATA |
|  | M2- OR: | CTTTGTTACCATCGGTACATTCTT |
| Secondary | S1fw: | GCTTATAATATGAGTATAAGGAGAA |
|  | N5rev: | CTGAAGAGGTACTGGTAGA |
|  | M5rev: | GCATTGCCAGAACTTGAA |
