# Supplementary material for: Large Variations in Malaria Parasite Carriage by Afebrile School Children Living in Nearby Communities in the Central Region of Ghana
Source: J Trop Med. 2020 Sep 22;2020:4125109. doi: 10.1155/2020/4125109 (PMC7528039; doi:10.1155/2020/4125109)
Supplement: Supplementary Materials — Additional file Table S1: primer details. Information on all the primers used in the study. Additional file Figure S1: representative images of P. falciparum, P. malariae, P. ovale, and P. vivax PCR products. Additional file Table S2: details of the false-positive samples. [file 4125109.f1.zip › 4125109.f1/31.8.2020 Sch screen Fig S1.pdf]

Supplementary file Figure S1. Representative images of *P. falciparum* , *P. malariae*, *P. ovale* and *P. vivax* PCR products

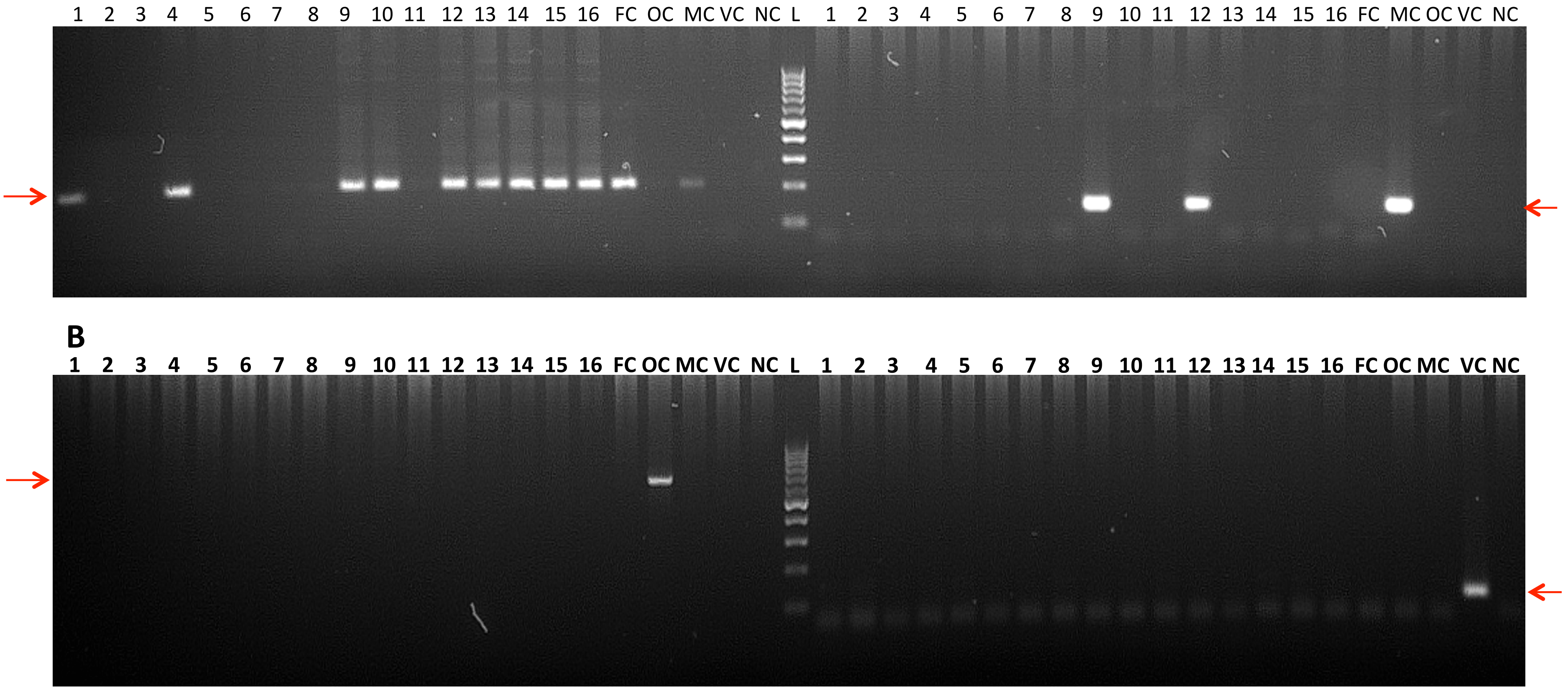

A: Left, *P. falciparum* (205 bp); Right, *P. malariae* (144 bp). **B**: Left, P. ovale (787 bp); Right, P. vivax (117 bp)
L, 100 bp ladder, with the lowest band representing 100 bp and the highest 1000 bp, with each band being 100 bp greater than the band below it.
Lanes **1-16** are representative samples from children from Kuful; **NC** is the negative control (water); **PC** is gDNA from 3D7 (MRA-102, *P. falciparum control*); **OC** is gDNA from a *P. ovale* field isolate; MC is gDNA from a Pf/Pm mixed infection (field isolate) used as an in-house P. mal control; VC is gDNA from *P. vivax* (obtained as part of the 2019 WHO NAAT EQA Scheme)
